# Defining When Nusinersen Starts to Work: Time to Clinical Benefit in Patients with SMA Types 1–3 from a Real-World Cohort in China

**DOI:** 10.3390/diagnostics16121828

**Published:** 2026-06-12

**Authors:** Ying Wu, Shuang Li, Yanbin Fan, Yuan Wu, Jie Zhang, Hui Dong, Yao Zhang, Xiaoling Yang, Hui Xiong, Cuijie Wei

**Affiliations:** 1 Children’s Medical Center, Peking University First Hospital, Beijing 102699, China; 2 Neurology Department, Beijing Children’s Hospital, Capital Medical University, Beijing 100045, China

**Keywords:** spinal muscular atrophy, Nusinersen, efficacy, safety, real world

## Abstract

**Background**: 5q spinal muscular atrophy (SMA) is a hereditary neuromuscular disorder characterized by progressive muscle weakness. Nusinersen, the first disease-modifying therapy for SMA, has demonstrated efficacy in both clinical trials and real-world studies. However, the precise timing of therapeutic onset following Nusinersen administration remains unclear. **Methods**: This retrospective study analyzed clinical data from patients with genetically confirmed 5q SMA who received Nusinersen treatment for at least six months at Peking University First Hospital. Motor function was assessed using standardized scales prior to each dose. **Results**: In total, 74 patients were screened, of whom 62 were enrolled, including 14 with type 1, 29 with type 2, and 19 with type 3 SMA. Thirty-two patients completed motor function assessments. After six months of treatment, 62.5% achieved a primary clinically meaningful response (an increase of ≥4 points in CHOP-INTEND or ≥3 points in HFMSE). Seven patients (21.9%) attained or regained motor milestones. Median improvements were 6 points in CHOP-INTEND (*p* = 0.001), 4 points in HFMSE (*p* = 0.003), and 1.5 points in RULM (*p* = 0.045). Further analysis indicated that the available median time to treatment response was approximately 2 months. In patients with severe scoliosis or prior spinal surgery, ultrasound-guided lumbar puncture demonstrated a high success rate (94.9%). Regarding safety, intrathecal injection-related adverse events occurred in eight patients (12.9%), and no adverse events led to treatment discontinuation. **Conclusions**: During the loading phase, Nusinersen provides clinical benefit for the majority of patients, with a median time to therapeutic response for monitoring of approximately 2 months. Ultrasound-guided intrathecal administration is the preferred approach for individuals with complicated spinal conditions. These findings may help guide clinical expectations for physicians, patients, and caregivers.

## 1. Introduction

5q Spinal muscular atrophy (SMA) is an autosomal recessive disorder characterized by progressive and symmetrical muscle weakness and atrophy [1]. It is caused either by a homozygous deletion of exon 7 or by compound heterozygous pathogenic variants in the survival motor neuron 1 (*SMN1*) gene [2]. The prevalence in the general population is approximately 1~2 /100,000 individuals [3]. Copy numbers of the highly homologous survival motor neuron 2 (*SMN2*) gene are correlated with disease severity [4]. Traditionally, based on the age of onset, motor milestones achieved and *SMN2* copy numbers, SMA is classified into four types: type 1 (infantile), type 2 (intermediate), type 3 (juvenile) and type 4 (adult) [1]. With the application of disease-modifying drugs, patients are recommended for reclassification based on motor function status [5].

Nusinersen, as the first drug approved for SMA [6], has had its long-term efficacy and safety well established through clinical trials and real-world data [7,8,9]. However, research focusing on pediatric patients with SMA type 1–3 observed that some subjects experienced a decline in motor scales after Nusinersen [10]. In addition, according to limited data presenting short-term efficacy, one study demonstrated that clinically meaningful improvements in motor function could be achieved after the loading dose [11], whereas such outcomes were found for only some patients in another cohort [12]. Further, the precise timing of therapeutic onset following Nusinersen administration has not been explored in previous studies. In this retrospective single-center study, motor status-stratified scales were used to characterize the timing of clinical benefit from Nusinersen in children with SMA.

## 2. Materials and Methods

### 2.1. Study Design

This retrospective single-arm observational study was conducted at a single center in Beijing, China. Nusinersen was administered via intrathecal injection, with loading doses given on days 1, 14, 28, and 63, followed by maintenance doses administered every four months. All subjects received Nusinersen for at least six months, with motor function assessments performed during the 24 h period prior to each dose. This time point was set to align with clinical practice and establish a uniform evaluation schedule.

### 2.2. Population

Patients with 5q SMA treated with Nusinersen at Peking University First Hospital between 1 August 2019 and 31 December 2022 were recruited. The inclusion criteria were as follows: (1) diagnosis of SMA based on clinical manifestations and confirmed pathogenic variants in *SMN1*; (2) age under 18 years at the time of first Nusinersen administration; (3) treatment duration of at least 6 months; and (4) written informed consent provided by the patients and their legal guardians. The exclusion criteria included (1) incomplete clinical data; (2) prior or current exposure to other disease-modifying therapies, such as Risdiplam or Zolgensma; and (3) coexisting conditions affecting motor function. SMA types 1–3 were defined according to the established clinical consensus [13].

### 2.3. Administration of Nusinersen

For patients without severe spinal deformity, a lumbar puncture was performed directly. For those with severe scoliosis or a history of spinal surgery, ultrasound-guided puncture was used as the first choice. If no feasible intervertebral access was identified under real-time ultrasound, computed tomography (CT)-guided puncture was used as an alternative. Patients were observed for 4 h after each procedure.

### 2.4. Study Outcomes

Baseline data were collected at enrollment, including demographics, clinical history, physical examination, and genetic testing results. In the genetic strategy, multiplex ligation-dependent probe amplification (MLPA) was first applied to detect the copy number of the *SMN1* and *SMN2* gene. For cases with SMA suspected clinically and only one copy of *SMN1*, Sanger sequencing was performed to identify microvariants.

### 2.5. Efficacy Outcomes

Motor function was assessed prior to each Nusinersen administration, using appropriate scales. Participants were stratified according to motor function prior to the first dose. For our patients, those unable to sit unassisted (defined as sitting independently for at least 10 s) were assessed using the Children’s Hospital of Philadelphia Infant Test of Neuromuscular Disorders (CHOP-INTEND). Participants able to sit independently were evaluated using the Hammersmith Functional Motor Scale Expanded (HFMSE), and the Revised Upper Limb Module (RULM) was administered conditionally when clinically practicable. Therapeutic response was defined as an improvement from the baseline of ≥4 points in CHOP-INTEND, ≥3 points in HFMSE, or ≥2 points in RULM, based on previously established clinically meaningful change thresholds in SMA [14,15,16,17]. Primary outcomes included changes in scores and the proportion of participants achieving a treatment response in CHOP-INTEND and HFMSE after 6 months of Nusinersen. In RULM, for reflecting upper limb function, the changes in scores and the proportion of responders were regarded as a secondary indicator. The time to therapeutic response in CHOP-INTEND and HFMSE was also evaluated and defined as the interval from the first dose of Nusinersen to the first documented improvement meeting the response criteria. In addition, exploratory analyses were conducted to identify potential baseline factors associated with improvements in motor function.

### 2.6. Safety Outcomes

Safety assessments included routine blood tests (complete blood count, biochemistry, and coagulation parameters) and cerebrospinal fluid (CSF) analysis (routine and biochemical examinations). Caregivers completed standardized questionnaires after each administration. All adverse events were reviewed by neurologists, and causality was evaluated.

### 2.7. Statistics Analysis

Descriptive statistics were used to summarize the baseline characteristics. Continuous variables are presented as the median (range) and categorical variables as frequencies (%). The Wilcoxon signed-rank test assessed changes in motor function scale scores from baseline to 6 months after Nusinersen administration. The Mann–Whitney U test was used for between-group comparisons of continuous variables, and Fisher’s exact test was used for categorical variables. Bonferroni correction was applied between different scales and subgroups. Spearman correlation analysis and multivariate binary logistic regression mode evaluated associations between therapeutic response in motor scale and baseline variables. The Kaplan–Meier method was applied to estimate the time to treatment response. Censoring criteria were formulated as follows: patients were censored if they were lost to follow-up, withdrew voluntarily from the study due to non-treatment-related reasons, or completed the entire follow-up period without achieving treatment response. The analysis of motor function scales focused on patients with complete data, and further sensitivity analyses were performed in all the patients with baseline data of motor scales. Missing data were addressed by multiple imputation and delta-adjusted multiple imputation with a downward adjustment of 4 points based on the clinically meaningful change in CHOP-INTEND and HFMSE. All analyses were performed using SPSS version 27.0, with a two-sided *p*-value < 0.05 considered statistically significant.

## 3. Results

### 3.1. Participants and Baseline Information

**Figure 1:**
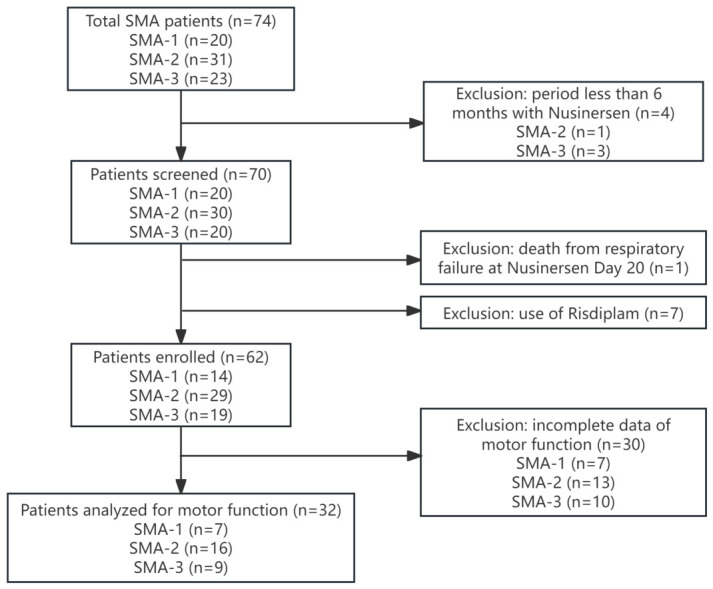
Flow diagram of screening process. SMA = spinal muscular atrophy.

The patient enrollment process is illustrated in Figure 1. A total of 74 patients received Nusinersen at our center during the study period. Twelve patients were excluded due to a short treatment duration or concomitant use of Risdiplam. Two patients with type 1 died of respiratory failure after 20 days and 14 months of treatment, respectively. Ultimately, 62 of 74 patients (83.8%) were included in the final analysis, including 14 patients (22.6%) with type 1, 29 (46.8%) with type 2, and 19 (30.6%) with type 3. Baseline characteristics are summarized in Table 1. The baseline characteristics between enrolled participants with complete motor scale data and those with missing data were compared, showing no significant statistical difference (Table S1).

### 3.2. Motor Function Outcomes

**Figure 2:**
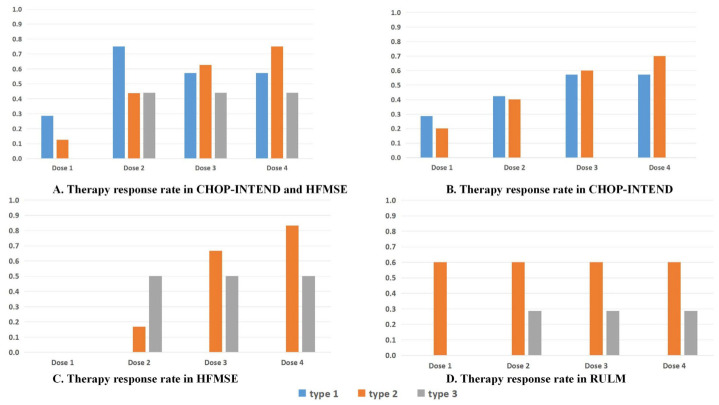
Results of therapy response rate during 6 months of Nusinersen. (**A**) Therapy response rate in CHOP-INTEND and HFMSE; (**B**) Therapy response rate in CHOP-INTEND; (**C**) Therapy response rate in HFMSE; (**D**) Therapy response rate in RULM. CHOP-INTEND = Children’s Hospital of Philadelphia infant test of neuromuscular disorders; HFMSE = Hammersmith functional motor scale expanded; RULM = the Revised Upper Limb Module.

After six months of Nusinersen treatment, four patients gained or regained independent sitting, and three cases with type 2 achieved independent standing. A total of 32 out of 62 patients (51.6%) completed motor function assessments (Table 2, Figure 2 and Figure S1), including seven patients with type 1, sixteen with type 2, and nine with type 3. Among 18 non-sitters, the median baseline CHOP-INTEND score was 31 (range 2–47), with a median increase of 6 points (range 0–15) at 6 months (*Z* = −3.419, *p* = 0.001); here, 64.7% met the predefined criteria for therapeutic response. Among 14 sitters/ walkers with HFMSE, the median baseline score was 27 points (range 10–62), with a median improvement of 4 points (range 0–10; *Z* = −3.190, *p* = 0.003); the therapy response rate was 64.3%. RULM was performed in 12 patients, showing a median increase of 1.5 points (range −1–6; *Z* = −2.442, *p* = 0.045). In total, after six months of treatment, 87.5% of patients showed increased scores in CHOP-INTEND and HFMSE, and 62.5% achieved therapy response (Table 2). Sensitivity analyses demonstrated response rates of 57.7%, 73.1%, and 65.4% for the original data, multiply imputed data, and delta-adjusted multiple imputation data with a 4-point reduction, respectively. No deviation between all the imputed data and original data was statistically significant, as zero fell within the 95% CI of response rate discrepancies (Table S2). In the exploratory subgroup analysis, we observed a statistically significant increase in CHOP-INTEND for patients with SMA type 2 (*p* = 0.016) (Table S3). With prolonged treatment, the motor function remained stable or continued to improve in most patients (Figure S2). Overall, these findings demonstrated that the majority of patients benefited in motor function during the six-month loading phase.

**Figure 3:**
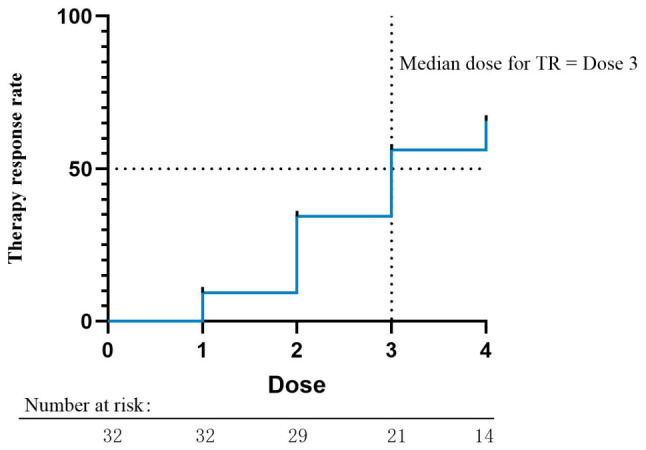
Therapy response rate in CHOP-INTEND and HFMSE during 6 months of Nusinersen. The blue line represents the cumulative therapy response rate across dose. The dotted line indicates the median therapy response rate was achieved at Dose 3, approximately 2 months. TR = therapy response.

To further evaluate the measurable time point to take effect for Nusinersen, survival analysis was performed. Patients who achieved a therapy response in CHOP-INTEND or HFMSE were considered to have reached the endpoint. The results revealed that the available median time to response occurred approximately 2 months after treatment initiation (Figure 3). Sensitivity analyses for the patients with baseline data of motor scales supported this finding (Figure S3).

Additional analyses were performed to explore associations between the baseline characteristics and therapeutic response at 6 months. Restricted by insufficient cases, univariate exploratory assessment presented that the disease duration was associated with RULM improvement (*Z* = −2.684, *p* = 0.007), whereas the *SMN2* copy number and baseline motor function were associated with changes in HFMSE (*p* = 0.014, *p* = 0.037 respectively). Baseline features related to CHOP-INTEND improvement were not observed (Tables S4–S7). Disease duration and *SMN2* copy number were incorporated into the final binary regression model. We observed that a ≤2 *SMN2* copy number seemed to be a trend toward poorer treatment response (OR = 0.186); however, this difference was not statistically significant (*p* = 0.055) (Table S8).

### 3.3. Respiratory and Feeding Support

Among the six patients receiving daily noninvasive ventilation (NIV) at baseline, three (50%) discontinued NIV after 6 months of Nusinersen treatment, including two with SMA type 1 and one with type 2. One patient continued ventilation for preventive purposes. Among two patients with tracheotomy, one died of causes unrelated to Nusinersen after 14 months of therapy, while the other remained tracheotomy-dependent due to clinical necessity. Although the number of patients requiring tube feeding did not decrease, caregivers of patients with SMA type 1 reported faster oral feeding and improved swallowing efficiency.

### 3.4. Intrathecal Injection Procedure

**Figure 4:**
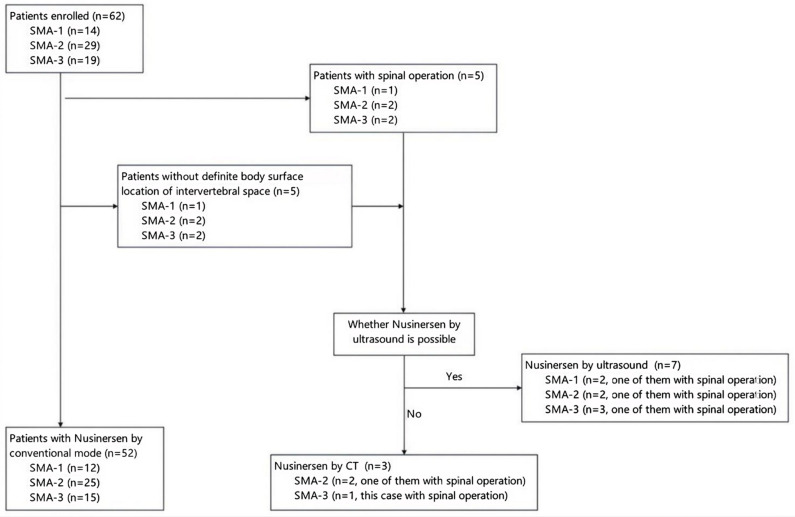
Flow diagram of intrathecal injection procedure. SMA = spinal muscular atrophy; CT = computerized tomography. Conventional mode means that the operation was performed by body surface positioning.

Fifty-two patients (83.9%) received intrathecal administration under surface landmark guidance. Ten patients with spine deformities or prior spinal surgery required imaging-assisted procedures (Figure 4), including ultrasound guidance in seven cases and CT guidance in three. A total of 403 intrathecal injections were performed. Of these, 39 were ultrasound-guided (success rate 94.9%), and 16 were CT-guided (success rate 100.0%).

### 3.5. Safety Profile

Adverse events related to intrathecal injection occurred in 8 of 62 patients (12.9%), including puncture site pain (6/8), nausea or vomiting (5/8), and dizziness or headache (5/8). Three patients developed transient fever after administration, which was cured within 72 h. One patient with SMA type 2 presented with high-normal baseline ALT (45 IU/L) due to non-viral fatty liver. After treatment initiation, intermittent elevations of ALT/AST (Maximum 106/56 IU/L) occurred, requiring hepatoprotection therapy until the sixth dose. The liver function subsequently remained stable without further intervention. Another patient with SMA type 2 showed transient CSF leukocytosis (15 cells/mm^3^, including 10 neutrophils) at the fifth dose, with normal CSF biochemistry and negative cultures; this resolved spontaneously. No treatment interruptions or permanent discontinuations occurred.

## 4. Discussion

In the present cohort, we included SMA patients with varying clinical severity and systematically evaluated changes in motor function during the first 6 months of treatment. Following the loading phase of Nusinersen, 62.5% of patients got a clinical response by month 6, and the measured median time to response is approximately 2 months. In addition, adverse events were generally mild, reversible and tolerable. Our findings provide additional real-world evidence regarding the early treatment response to Nusinersen in patients with SMA.

In our cohort, we characterized early motor function changes during the first six months of treatment. A cumulative therapy response rate of 62.5% in CHOP-INTEND and HFMSE was observed, consistent with the 53–72% reported in previous studies [11,15,18,19,20]. These responses represent clinically meaningful improvements in SMA. For non-sitters, such improvements may reflect gains in active antigravity motor capacity, whereas for sitters they may translate into improved sitting stability and transfer function, thereby reducing caregiver burden [21,22,23]. At the 6-month dose of Nusinersen, the response rate in HFMSE was higher than RULM, and during the loading phase, we observed a progressive increase in HFMSE for patients with type 2, different from the stable rate in RULM. Similar findings were presented in a study including short-term data: clinically meaningful improvements in HFMSE were observed after six months, whereas RULM failed and stayed relatively stable [18]. For sitters, relativity stable scores in RULM suggested the retention of upper limb function, allowing them to perform self-care tasks including eating and hair grooming autonomously. As reported in earlier research, the RULM scale suffers from a ceiling effect among individuals with well-preserved upper limb function, failing to capture slight improvements effectively [18,24]. Given the relatively high median score of 30.5 points in our patients, the unremarkable improvement in RULM compared with HFMSE may be explained by the ceiling effect. The application of composite functional scales and extended long-term follow-up are likely to identify additional therapeutic benefits [25].

Furthermore, Kaplan–Meier analysis showed that the median time to treatment response for measure was approximately 2 months. Actually, limited research has shown the results of motor function during the loading phase, and inconsistent findings have been observed. A study from China reported significant improvements in CHOP-INTEND at Day 60 and Day 180 for HFMSE after initial Nusinersen [26]. Another study from northwestern Iran presented similar improvements in HFMSE at two weeks [18]. These studies differed substantially in patient characteristics and outcome assessment methods [18,26]: both the Chinese cohort and our study recruited SMA type 1–3 patients, including those with severe complications; in contrast, the Iranian cohort solely enrolled patients with SMA type 2 and 3 with superior baseline motor performance and defined a change of ≥2 points in HFMSE as the minimal clinically important difference. More importantly, although previous studies reported motor outcomes at selected follow-up visits, they did not directly evaluate the time to treatment response using a predefined response criterion and time-to-event analysis. By applying a standardized response definition and Kaplan–Meier analysis, our study provides an estimate of the timing of treatment response based on scheduled assessments during the loading phase. The estimated median time to response of approximately 2 months may provide a useful reference for planning early therapeutic evaluation in clinical practice.

We additionally performed exploratory analyses to identify baseline predictors of treatment response. Our preliminary results suggested that having more than two *SMN2* copy numbers was potentially related to better therapy response, and the disease duration had no significant correlation with the response. According to a systematic review [27], disease duration before administration has been reported as a strong predictor, and the *SMN2* copy number serves as a favorable predictive factor for presymptomatic patients. Such discrepancies may be explained by our relatively small sample size, which limited the statistical power of the regression models, as well as potential confounding factors that were not fully accounted for.

Dyspnea is common in SMA type 1 and weaker SMA type 2. Once a diagnosis of SMA type 1 is established, a household ventilator and cough assistant machine is generally recommended [28]. The available evidence suggests Nusinersen can delay the need for ventilators or permanent ventilation, but it does not appear to substantially alter the ultimate respiratory outcome in most patients [14,29,30,31,32]. In our cohort, however, 50% (3/6) of patients were able to discontinue non-invasive ventilation during follow-up. Notably, all of them required respiratory support at baseline, attributed to a clinical measure for acute respiratory illness. The potential impact of Nusinersen on SMA-related respiratory deterioration remains undetermined in the present cohort. Dysphagia represents a non-negligible challenge in non-ambulant patients with SMA [33]. In our cohort, no significant improvement in feeding status was observed, which is consistent with previous findings [10,34,35].

Nusinersen requires repeated intrathecal administration because it cannot cross the blood–brain barrier [36]. However, intrathecal injection using the traditional posterior interlaminar approach can be challenging in patients with severe scoliosis or prior spinal surgery. Various image-guided techniques have been explored [37,38], including fluoroscopy, CT, ultrasound guidance; subcutaneous systems such as intrathecal port and Ommaya reservoirs have also been applied [39]. At our center, ultrasound-guided intrathecal injection through the interspinous plane was adopted as the first-line approach for these technically difficult patients [40], achieving a success rate of 94.9%. For the few unsuccessful cases, CT-guided puncture was subsequently performed, resulting in an overall success rate of 100%. Based on our experience, ultrasound guidance may serve as the preferred initial approach given its lack of radiation exposure, while CT guidance represents an effective alternative when ultrasound guidance fails.

The incidence of post-lumbar puncture syndrome in our study was lower than previously reported [41], which may be attributable to standardized lumbar puncture procedures. Two adverse events were considered possibly related to Nusinersen, including aseptic meningitis and transient liver function abnormalities. The aseptic meningitis resolved without sequelae, while the liver enzyme abnormality was mild and reversible with hepatoprotective treatment. Notably, the patient had pre-existing fatty liver, which may represent a potential risk factor. Overall, no adverse reactions led to treatment discontinuity, indicating good treatment persistence.

Nevertheless, several limitations should be acknowledged. Firstly, as a retrospective real-world investigation about a rare disease, this study has inherent limitations in sample size and clinical data collection. Although no significant differences were found in the baseline characteristics between enrolled participants and those with missing data, potential differences in disease progression and caregiver burden between the two groups could not be assessed with the available dataset. While the sensitivity analyses supported our findings, missing motor function assessments in a subset of patients may still have introduced bias and reduced the strength of the conclusions. In addition, owing to the non-continuous monitoring of motor function, it was impossible to clarify the specific time to exert efficacy based on existing information. The 2-month interval herein is identified a feasible window for monitoring therapeutic effects. Optimized prospective designs with a larger size and continuous follow-up will be helpful. Moreover, we acknowledge the potential risk of type I error inflation due to multiple comparisons and subgroup analyses; therefore, findings from subgroup and predictor analyses should be interpreted as exploratory and hypothesis-generating rather than confirmatory.

## 5. Conclusions

Our study supports the short-term efficacy and safety of Nusinersen in Chinese children with SMA types 1–3. Motor function improved in most patients after six months of treatment, with an estimated median time to clinical response of approximately two months. Treatment-related adverse events were generally mild and well tolerated. For cases with challenging spinal conditions, ultrasound-assisted intrathecal puncture is prioritized clinically. These findings may help guide clinical expectations for physicians, patients, and caregivers. Notably, they should be interpreted within the context of the relatively small sample size, missing motor-function assessments in a subset of patients, and the exploratory nature of the predictor analyses.

## Figures and Tables

**Table 1:** Baseline characteristics of SMA patients with only Nusinersen for more than 6 months.

	All Patients	SMA Type 1	SMA Type 2	SMA Type 3
*n*	62	14	29	19
Male sex, *n* (%)	25 (40.3)	6 (42.9)	11 (37.9)	8 (42.1)
*SMN2* copy number, *n*	53	12	24	17
2, *n* (%)	9 (17.0)	7 (58.3)	2 (8.3)	0
3, *n* (%)	39 (73.6)	5 (41.7)	22 (91.7)	12 (70.6)
4, *n* (%)	5 (9.4)	0	0	5 (29.4)
Age at onset, months, median (min–max)	11.5 (0–139)	3.0 (0–6)	10.0 (6–19)	16.0 (8–139)
Age at baseline, months, median (min–max)	63.0 (4–199)	22.5 (4–165)	90.0 (17–198)	55.0 (25–199)
Disease duration at baseline, months, median (min–max)	49.0 (2–186)	18.5 (4–160)	75 (2–186)	40.0 (7–183)
Treatment duration, months, median (min–max)	11.0 (7–47)	11.0 (7–41)	11.0 (8–38)	11.0 (8–47)
Optimal motor function at baseline, *n* (%)				
Unable to sit independently	21 (33.9)	14 (100)	6 (20.7)	1 (5.3)
Sitting independently	26 (41.9)	NA	23 (79.3)	3 (15.8)
Standing independently	NA	NA	NA	NA
Walking independently	15 (24.2)	NA	NA	15 (78.9)
Respiratory support, *n* (%)				
NIV ≤ 16 h/d	6 (9.7)	3 (21.4)	3 (10.3)	NA
NIV > 16 h/d	NA	NA	NA	NA
Tracheotomy	2 (3.2)	2 (14.3)	NA	NA
Nasal feeding, *n* (%)	2 (3.2)	2 (14.3)	NA	NA
Spinal surgery, *n* (%)	5 (8.1)	1 (7.1)	2 (6.9)	2 (10.5)
Severe scoliosis (Cobb angle ≥ 50°), *n* (%)	5 (8.1)	4 (28.6)	1 (3.4)	NA

SMA = spinal muscular atrophy; SMN = survival motor neuron; NA = not applicable; NIV = non-invasive ventilation; h/d = hours/day; Cobb angle—the angle between the upper endplate of the upper end vertebrae and the lower endplate of the lower end vertebrae on coronal plane, which reflects the severity of scoliosis.

**Table 2:** Scores of motor function scales at baseline and after 6 months of treatment with Nusinersen.

Motor Scale	N	Median Score ^a^ at Baseline	Median Score ^a^ at Nusinersen for 6 Months	Median Differences ^b^	Therapy Response N (%)	*Z*	*p*	Bonferroni-Adjusted *p* *
CHOP-INTEND	18	31.0 (2–47)	38.5 (2–55)	6.0 (0–15)	11 (61.1)	−3.419	0.001	NA
HFMSE	14	27.0 (10–62)	32.5 (15–64)	4.0 (0–10)	9 (64.3)	−3.190	0.001	0.003
RULM	12	30.5 (8–37)	32.5 (14–37)	1.5 (−1–6)	5 (41.7)	−2.442	0.015	0.045

CHOP-INTEND = Children’s Hospital of Philadelphia infant test of neuromuscular disorders; HFMSE = Hammersmith functional motor scale expanded; RULM = the Revised Upper Limb Module; NA = not applicable. Therapy response refers to the increase of ≥4 points in CHOP-INTEND, ≥3 points in HFMSE or ≥2 points in RULM.; ^a^: Data are the median score (min–max). ^b^: Data are the median differences (min–max) for 6 months of treatment versus baseline. * *p* value was assessed by the Wilcoxon signed-rank test and Bonferroni-adjusted. A bilateral *p*-value <0.05 was considered statistically significant.

## Data Availability

The data presented in this study are available on request from the corresponding author due to privacy.

## Supplementary Materials

The following supporting information can be downloaded at https://www.mdpi.com/article/10.3390/diagnostics16121828/s1, Figure S1: Data pattern of motor function assessments in CHOP-INTEND and HFMSE during follow-up; Figure S2: Results of motor function assessment at 6 months of Nusinersen; Figure S3: Therapy response rate in CHOP-INTEND and HFMSE during 6 months of Nusinersen for patients with baseline scores; Table S1: Baseline characteristics of SMA patients with and without complete motor data; Table S2: Sensitivity analyses on therapy response in CHOP-INTEND and HFMSE at 6 months of Nusinersen; Table S3: Scores of motor function scales at baseline and after 6 months of Nusinersen in different clinical types; Table S4: Potential effect factors on therapy response at 6 months of Nusinersen; Table S5: Potential effect factors on therapy response of CHOP-INTEND at 6 months of Nusinersen; Table S6: Potential effect factors on therapy response of HFMSE at 6 months of Nusinersen; Table S7: Potential effect factors on therapy response of RULM at 6 months of Nusinersen; Table S8: Results of multifactorial logistic analysis with potential factors on therapy response at 6 months of Nusinersen.

## Abbreviations

The following abbreviations are used in this manuscript:

SMA	Spinal muscular atrophy
*SMN1*	Survival motor neuron 1
*SMN2*	Survival motor neuron 2
CT	Computerized tomography
CHOP-INTEND	Children’s Hospital of Philadelphia Infant Test of Neuromuscular Disorders
HFMSE	Hammersmith functional motor scale expanded
RULM	the Revised Upper Limb Module
CSF	cerebrospinal fluid
NIV	non-invasive ventilation
