# Supplementary material for: Defining When Nusinersen Starts to Work: Time to Clinical Benefit in Patients with SMA Types 1–3 from a Real-World Cohort in China
Source: Diagnostics (Basel). 2026 Jun 12;16(12):1828. doi: 10.3390/diagnostics16121828 (PMC13297879; doi:10.3390/diagnostics16121828)
Supplement: Supplementary file 1 [file diagnostics-16-01828-s001.zip › diagnostics-4259885-supplementary.pdf]

SUPPLEMENTARY MATERIALS

Table S1. Scores of motor function scales at baseline and after 6 months of Nusinersen in different clinical types.

	n	Median score[a] at Baseline	Median score[a] at Nusinersen for 6 months	Median differences[b]	Therapy response n (%)	Z	p*
**CHOP-INTEND**							
Type 1	7	29.0 (2-47)	36.0 (2-47)	6.0 (0-15)	4 (57.1)	-2.032	0.084
Type 2	10	40.0 (27-46)	47.0 (37-55)	6.5 (0-15)	7 (70.0)	-2.668	0.016
Type 3	1	29	32	3	0 (0)		
**HMFSE**							
Type 2	6	21.0 (10-27)	24.5 (15-36)	5.0 (2-10)	5 (83.3)	-2.226	0.156
Type 3	8	48.0 (18-62)	49.5 (26-64)	2.5 (0-8)	4 (50.0)	-2.375	0.108
**RULM**							
Type 2	5	24.0 (8-37)	23.0 (14-37)	2.0 (-1-6)	3 (60.0)	-1.461	0.864
Type 3	7	32.0 (25-36)	35.0 (26-37)	1.0 (0-4)	2 (28.6)	-2.041	0.246

CHOP-INTEND = Children's Hospital of Philadelphia infant test of neuromuscular disorders; HFMSE = Hammersmith functional motor sca le expanded; RULM = the Revised Upper Limb Module; SMA = spinal muscular atrophy. Therapy response refers to the increase of ≥4 points in CHOP-INTEND, ≥3 points in HFMSE or ≥2 points in RULM. a: Data are median score (min–max). b: Data are median differences (min–max) for 6-month treatment versus baseline.* p value was assessed by the Wilcoxon signed-rank test and Bonferroni-adjusted. A bilateral p-value <0.05 was considered statistically significant.

Table S2. Sensitivity analyses on therapy response in CHOP-INTEND and HFMSE at 6 months of Nusinersen.

	n	Therapy response n (95% CI)	Therapy response rate % (95% CI)	Absolute difference % (95% CI)
Patients with complete data	32	20	62.5	NA
Patients with original data	52	30	57.7	-4.8
Patients with multiple imputation	52	38 (36, 41)	73.1 (69.2,78.8)	10.1 (-3.8, 24.1)
Patients with delta-adjusted multiple imputation				
Delta = -1	52	37 (35, 40)	71.2 (67.3, 76.9)	8.1 (-6.2, 22.6)
Delta = -2	52	36 (34, 39)	69.2 (65.4, 75.0)	6.3 (-8.3, 21.1)
Delta = -3	52	35 (33, 38)	67.3 (63.5, 73.1)	4.3 (-10.7,19.3)
Delta = -4	52	34 (32.5, 37)	65.4 (62.5, 71.2)	2.6 (-12.0, 17.5)

CHOP-INTEND = Children's Hospital of Philadelphia infant test of neuromuscular disorders; HFMSE = Hammersmith functional motor scale expanded; CI = confidence interval; NA = not applicable. Therapy response refers to the increase of ≥4 points in CHOP-INTEND or ≥3 points in HFMSE. Complete data included baseline motor scale scores and those obtained before each dose over 6 months of Nusinersen. Patients with original data refer to those with baseline data of motor scales. In delta-adjusted multiple imputation, the delta value represents the downward adjustment points for imputed values. Absolute difference refers to the deviation between the corresponding value and 62.5% (the therapy response rate from patients with complete data), and 95% confidence intervals were evaluated based on patient-level bootstrap.

Table S3. Potential effect factors on therapy response at 6 months of Nusinersen.

	Therapy response	No therapy response	Z	p*
n (%)	20 (62.5)	12 (37.5)		
Gender, n (%)			NA	0.473
Male	7 (53.8)	6 (41.2)		
Female	13(65.0)	6 (35.0)		
Age at onset, months, median (min–max)	20 (3-18)	12 .5(0.5-24)	-0.898	0.387
Age at baseline, months, median (min–max)	59 (17-199)	48.5 (9-187)	-0.642	0.526
Disease duration at baseline, months, median (min–max)	46 (2-183)	32.5 (9-176)	-0.798	0.425
Clinical classification, n (%)			NA	0.552
Type 1	4 (57.1)	3 (42.9)		
Type 2	12 (75.0)	4 (25.0)		
Type 3	4 (44.4)	5 (55.6)		
Copies of *SMN2*, n (%)			NA	0.001
2 copies	2 (50.0)	2 (50.0)		
3 copies	17 (85.0)	3 (15.0)		
4 copies	0	3 (100.0)		

NA = not applicable. Therapy response refers to the increase of ≥4 points in CHOP-INTEND or ≥3 points in HFMSE. * p value was assessed by the Mann–Whitney U test for continuous variables and Fisher's exact test for categorical variables, and a bilateral p-value <0.05 was considered statistically significant.

Table S4. Potential effect factors on therapy response of CHOP-INTEND at 6 months of Nusinersen.

	Therapy response	No therapy response	Z	p*
n (%)	11 (61.1)	7 (38.9)		
Gender, n (%)			NA	0.332
Male	3 (42.9)	4 (57.1)		
Female	8 (72.7)	3 (27.3)		
Age at onset, months, median (min–max)	7 (3-18)	10 (0.5-16)	-0.456	0.649
Age at baseline, months, median (min–max)	42 (19-150)	32 (9-187)	-0.498	0.618
Disease duration at baseline, months, median (min–max)	39 (3-147)	27 (9-176)	-0.498	0.618
Clinical classification, n (%)			NA	0.464
Type 1	4 (57.1)	3 (42.9)		
Type 2	7 (70.0)	3 (30.0)		
Type 3	0 (0)	1 (100.0)		
Copies of *SMN2*, n (%)			NA	0.520
2 copies	2 (50.0)	2 (50.0)		
3 copies	8 (80.0)	2 (20.0)		
Scores at baseline, points, median (min–max)	31 (15-45)	39 (2-47)	-0.455	0.659

CHOP-INTEND = Children's Hospital of Philadelphia infant test of neuromuscular disorders; NA = not applicable. Therapy response of CHOP-INTEND refers to no less than a four-point increase. * p value was assessed by the Mann–Whitney U test for continuous variables and Fisher's exact test for categorical variables, and a bilateral p-value <0.05 was considered statistically significant.

Table S5. Potential effect factors on therapy response of HFMSE at 6 months of Nusinersen.

	Therapy response	No therapy response	Z	p*
n (%)	9 (64.3)	5 (35.7)		
Gender, n (%)			NA	1.000
Male	4 (66.7)	2 (33.3)		
Female	5 (62.5)	3 (37.5)		
Age at onset, months, median (min–max)	15(6-18)	16 (11-24)	-1.075	0.282
Age at baseline, months, median (min–max)	63 (17-199)	53 (39-73)	-0.667	0.505
Disease duration at baseline, months, median (min–max)	52 (2-183)	35 (15-62)	-0.801	0.423
Clinical classification, n (%)			NA	0.301
Type 2	5 (83.3)	1 (16.7)		
Type 3	4 (50.0)	4(50.0)		
Copies of *SMN2*, n (%)			NA	0.014
3 copies	9 (90.0)	1 (10.0)		
4 copies	0 (0)	3 (100.0)		
Scores at baseline, points, median (min–max)	26 (10-49)	48 (24-62)	-2.080	0.037

HFMSE = Hammersmith functional motor scale expanded; NA = not applicable. Therapy response of HFMSE refers to no less than a three-point increase. * p value was assessed by the Wilcoxon signed-rank test, and a bilateral p-value <0.05 was considered statistically significant.

Table S6. Potential effect factors on therapy response of RULM at 6 months of Nusinersen.

	Therapy response	No therapy response	Z	p*
n (%)	5 (41.7)	7 (58.3)		
Gender, n (%)			NA	1.000
Male	2 (50.0)	2(50.0)		
Female	3 (37.5)	5 (62.5)		
Age at onset, months, median (min–max)	14 (10-24)	15 (6-18)	-0.327	0.744
Age at baseline, months, median (min–max)	44 (35-62)	73 (55-199)	-2.684	0.007
Disease duration at baseline, months, median (min–max)	30 (15-46)	62 (40-183)	-2.684	0.007
Clinical classification, n (%)			NA	0.558
Type 2	3 (60.0)	2 (40.0)		
Type 3	2 (28.6)	5 (71.4)		
Copies of *SMN2*, n (%)			NA	1.000
3 copies	3 (33.3)	6 (66.7)		
4 copies	2 ((66.7)	1 (33.3)		
Scores at baseline, points, median (min–max)	31 (8-37)	30 (24-36)	-0.244	0.808

RULM = the Revised Upper Limb Module; NA = not applicable. Therapy response of RULM refers to no less than a two-point increase. * p value was assessed by the Wilcoxon signed-rank test, and a bilateral p-value <0.05 was considered statistically significant.

Table S7 Results of multifactorial logistic analysis with potential factors on therapy response at 6 months of Nusinersen.

	β	Wald Chi-square	OR (95%CI)	p^*
Disease duration at baseline	-0.005	0.837	0.995 (0.984, 1.006)	0.360
Copies of *SMN2* (≤2 vs >2)	-1.684	3.676	0.186 (0.033, 1.038)	0.055

OR = odds ratio; CI = confidence interval. Therapy response refers to the increase of ≥4 points in CHOP-INTEND or ≥3 points in HFMSE. * p value <0.05 was considered statistically significant.

Table S8. Baseline characteristics of SMA patients with or without complete motor data.

	All patients	Patients with complete motor data	Patients without complete motor data	p*
n	62	32	30	NA
Male sex, n (%)	25 (40.3)	13 (40.6)	12 (40.0)	1.000
SMA type				
type 1, n (%)	14 (22.6)	7 (21.9)	7 (23.3)	0.891
type 2, n (%)	29 (46.8)	16 (50.0)	13 (43.3)	0.598
type 3, n (%)	19 (30.6)	9 (28.1)	10 (33.3)	0.657
SMN2 copy number, n	53	27	26	1.000
2, n (%)	9 (17.0)	6 (22.2)	5 (19.2)	0.911
3, n (%)	39 (73.6)	19 (70.4)	20 (76.9)	0.946
4, n (%)	5 (9.4)	2 (7.4)	1 (3.8)	0.520
Age at onset, months, median (min–max)	11.5(0-139)	10.5(0-24)	12(0-139)	0.563
Age at baseline, months, median (min–max)	63.0(4-199)	54.0(9-199)	71.5(4-198)	0.545
Disease duration at baseline, months, median (min–max)	49.0(2-186)	39.5(2-183)	53.5(2-186)	0.894
Treatment duration, months, median (min–max)	11.0(7-47)	11.0(8-40)	11.0(7-47)	0.498
Optimal motor function at baseline, n (%)				
Unable to sit independently	21 (33.9)	11 (34.4)	10 (33.3)	0.795
Sitting independently	26 (41.9)	15 (46.9)	11 (36.7)	0.307
Standing independently	NA	NA	NA	NA
Walking independently	15(24.2)	6 (18.8)	9 (30.0)	0.379
Respiratory support n (%)				0.431
NIV ≤16h/d	6 (9.7)	4 (12.5)	2 (6.7)	0.395

NIV ＞16h/d	NA	NA	NA	NA
Tracheotomy	2 (3.2)	2 (6.3)	0	0.147
Nasal feeding, n (%)	2 (3.2)	1 (3.1)	1 (3.3)	1.000
Spinal surgery, n (%)	5 (8.1)	3 (9.4)	2 (6.7)	1.000
Severe scoliosis (Cobb angle≥50°) n (%)	5 (8.1)	2 (6.3)	3 (10)	0.667

SMA = spinal muscular atrophy; SMN = survival motor neuron; NA = not applicable; NIV = noninvasive ventilation; h/d = hours/day; Cobb angle: the angle between the upper endplate of the upper end vertebrae and the lower endplate of the lower end vertebrae on coronal plane, which reflects the severity of scoliosis .

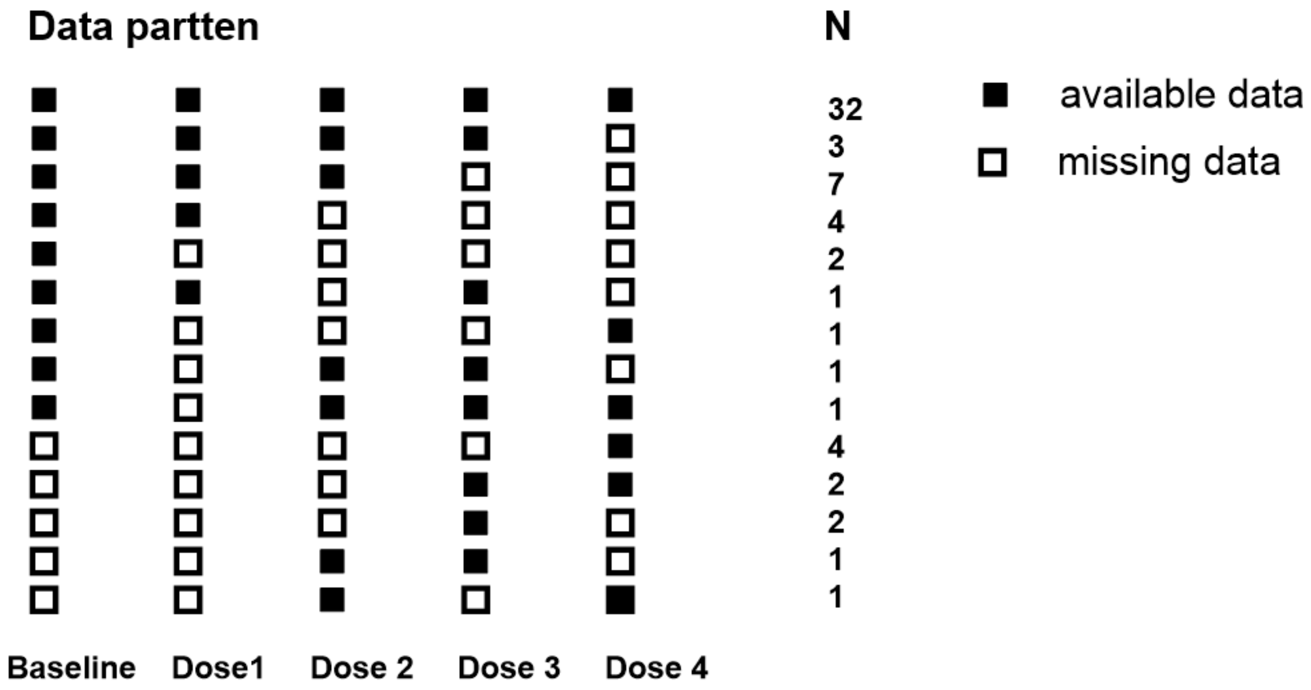

Figure S1. Data pattern of motor function assessments in CHOP-INTEND and HFMSE during follow-up.

Black squares indicate available data, and white squares indicate missing data. All patients were included in the analysis of baseline characteristics and changes in motor milestones. Patients with complete motor scale data contributed to primary outcome and survival analysis, and all subjects with valid baseline scores were incorporated into relevant sensitivity analyses.

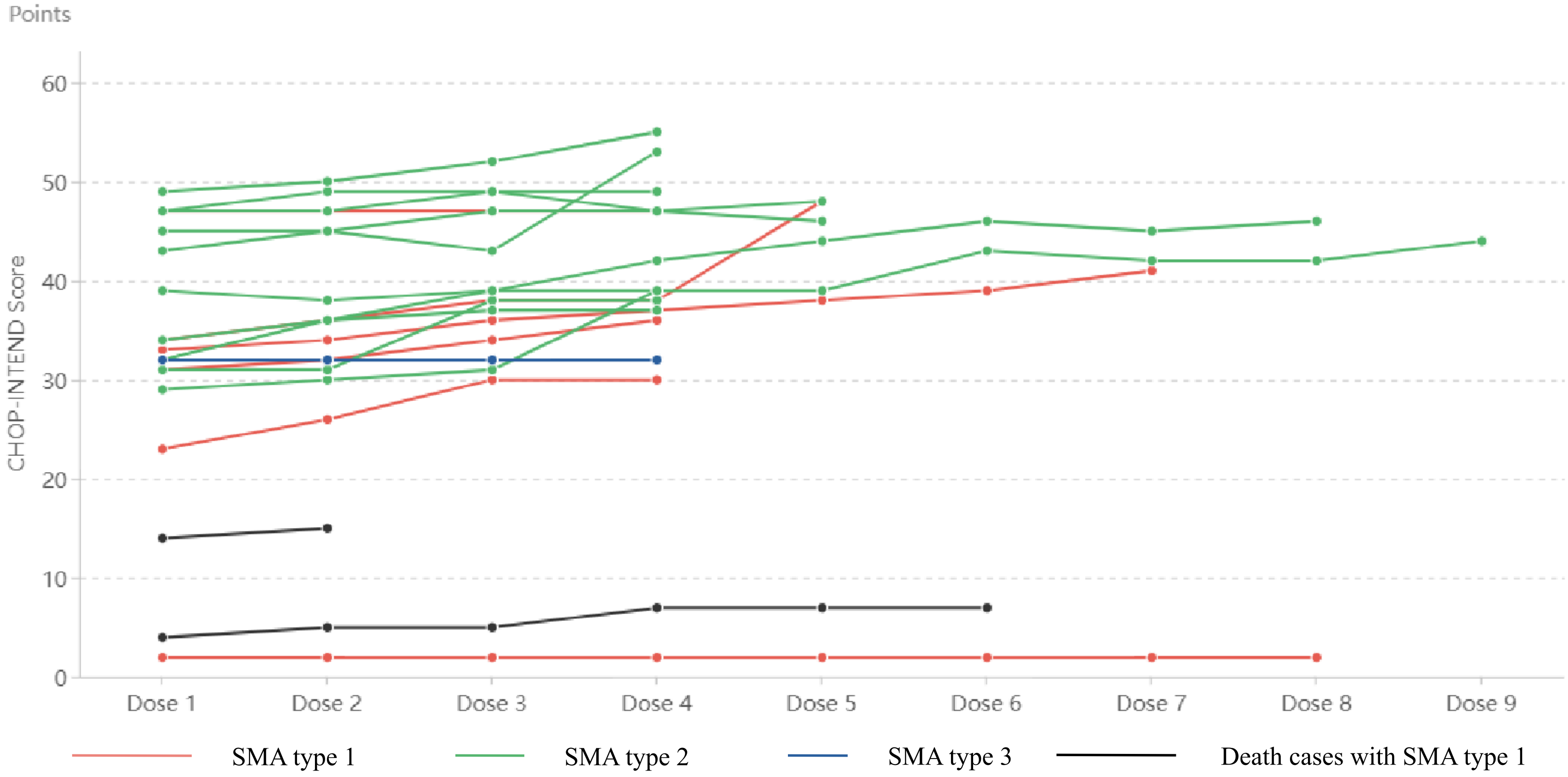

A. CHOP-INTEND score for each patient.

Two of patients with SMA type 1 died of respiratory problems, and one of the two died after 20 days of treatment when medical interventions were refused by the guardians.

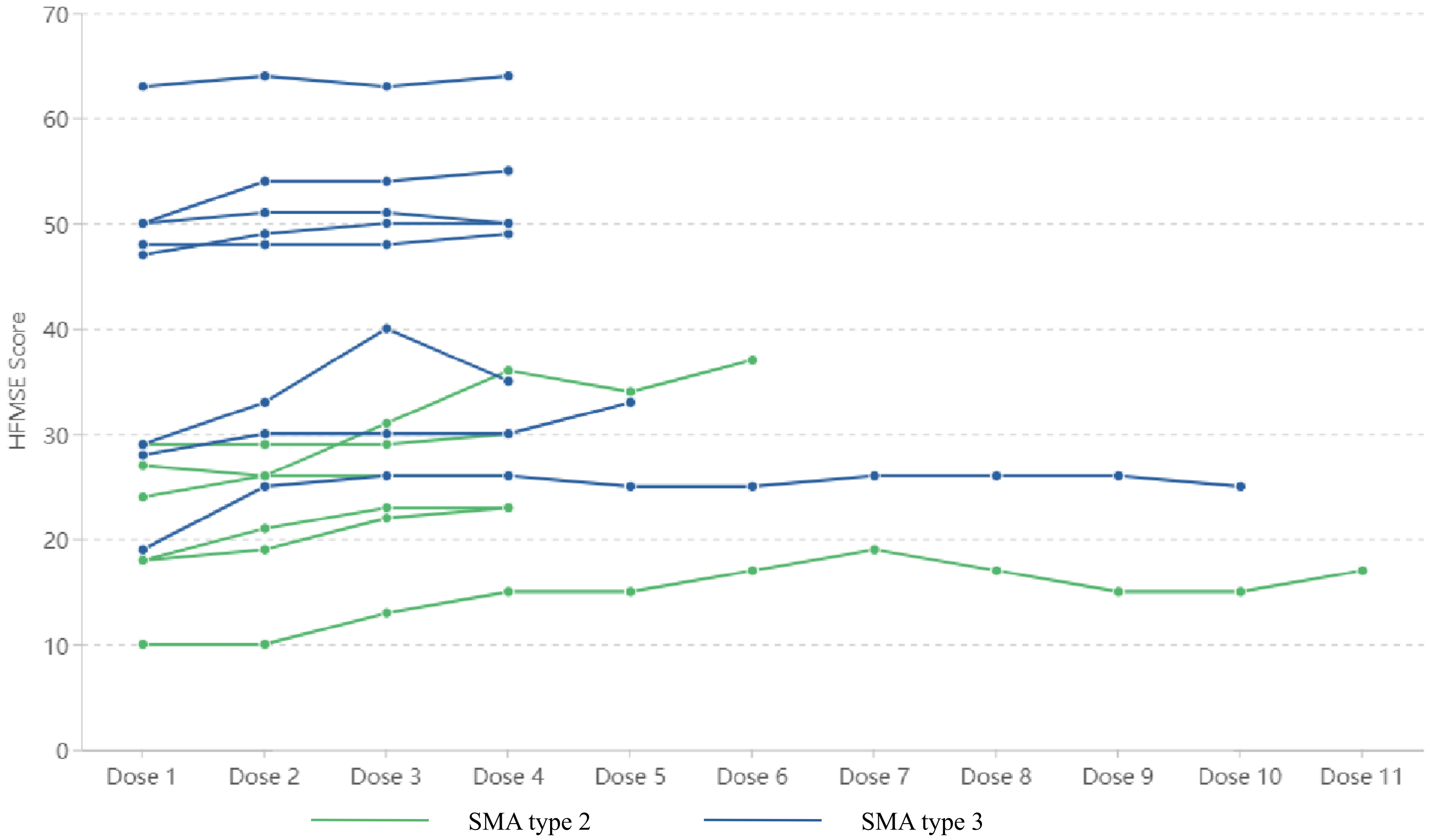

B. HFMSE score for each patient.

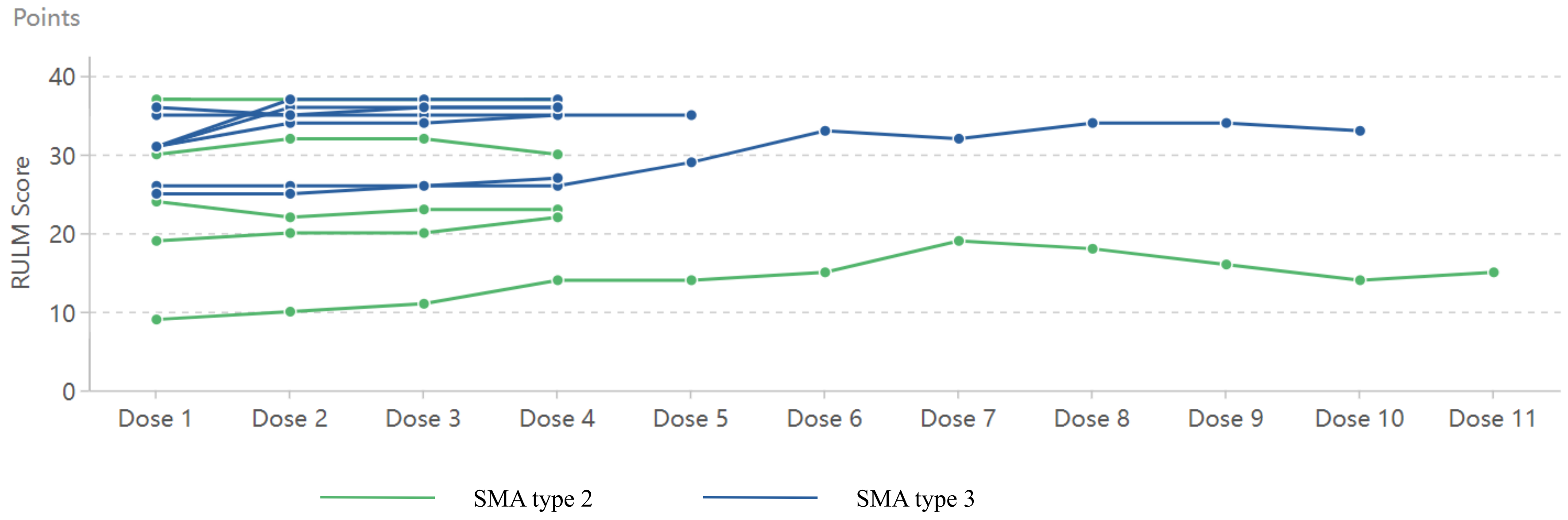

C. RULM score for each patient.

Figure S2. Results of motor function assessment at least 6 months of Nusinersen.

CHOP-INTEND = Children's Hospital of Philadelphia infant test of neuromuscular disorders; HFMSE = Hammersmith functional motor scale expanded; RULM = the Revised Upper Limb Module.

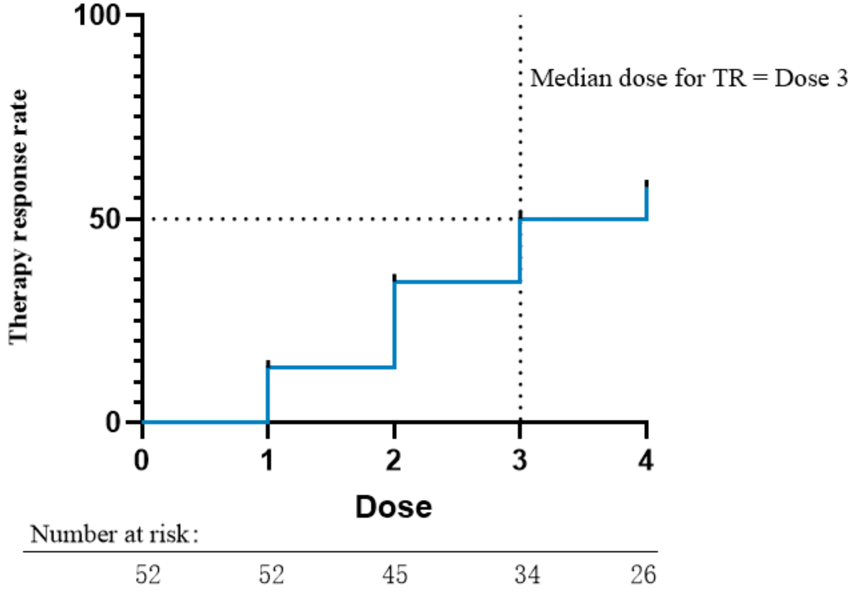

Figure S3. Therapy response rate in CHOP-INTEND and HFMSE during 6 months of Nusinersen for patients with baseline scores.

TR = therapy response. Survival analysis for the imputed dataset showed the time to median therapy response rate was also approximately 2 months.
